# Is There an Interest to Use Deuteron Beams to Produce Non-Conventional Radionuclides?

**DOI:** 10.3389/fmed.2015.00031

**Published:** 2015-05-11

**Authors:** Cyrille Alliot, Nadia Audouin, Jacques Barbet, Anne-Cecile Bonraisin, Valérie Bossé, Cécile Bourdeau, Mickael Bourgeois, Charlotte Duchemin, Arnaud Guertin, Ferid Haddad, Sandrine Huclier-Markai, Rabah Kerdjoudj, Johan Laizé, Vincent Métivier, Nathalie Michel, Marcel Mokili, Mickael Pageau, Aurélien Vidal

**Affiliations:** ^1^GIP Arronax, Saint-Herblain, France; ^2^CRCNA, CNRS, INSERM, Université de Nantes, Nantes, France; ^3^Subatech, EMN-IN2P3/CNRS, Université de Nantes, Nantes, France

**Keywords:** radionuclide production, deuteron, theranostic, copper-64, rhenium-186, scandium-44

## Abstract

With the recent interest on the theranostic approach, there has been a renewed interest for alternative radionuclides in nuclear medicine. They can be produced using common production routes, i.e., using protons accelerated by biomedical cyclotrons or neutrons produced in research reactors. However, in some cases, it can be more valuable to use deuterons as projectiles. In the case of Cu-64, smaller quantities of the expensive target material, Ni-64, are used with deuterons as compared with protons for the same produced activity. For the Sc-44m/Sc-44g generator, deuterons afford a higher Sc-44m production yield than with protons. Finally, in the case of Re-186g, deuterons lead to a production yield five times higher than protons. These three examples show that it is of interest to consider not only protons or neutrons but also deuterons to produce alternative radionuclides.

## Introduction

Radionuclides are used in nuclear medicine for both imaging and/or therapy (1). For the last two decades, positron emission tomography (PET) and molecular radiotherapy have developed rapidly. Fluorine-18 and especially F-18 fluorodeoxyglucose, FDG, is the main driving force for PET imaging (1) whereas yttrium-90 and lutetium-177 have shown promising clinical results for antibody or peptide labeling.

Recently, the concept of theranostics, which is a treatment strategy that combines therapy and diagnosis, has emerged. By doing imaging prior to the treatment, it is possible to select patients that will respond to a given treatment, to make dosimetry prior to therapy, and to define the activity to inject. Treatment efficacy may also be assessed. To achieve this aim, several types of isotopes can be selected: radionuclides that possess radiations for both imaging and therapy, as for example Sn-117m, pairs of radionuclide of the same element, like I-124 (for imaging) and I-131(for treatment), or radionuclides with comparable chemical properties like Tc-99m (for imaging) and Re-186g (for treatment).

The theranostic approach has renewed the interest for unconventional radionuclides in nuclear medicine and many radioisotopes with different properties (half-life, beta energy, gamma emissions) are under study for either imaging or therapeutic use. These isotopes are mostly produced using biomedical cyclotrons, which usually accelerate low-energy protons, or nuclear reactors, depending on the radiation of interest. In all cases, it is of great importance to get high-specific activity.

The aim of this paper is to illustrate the interest of using deuterons as projectiles to produce alternative radionuclides for medical applications. For that purpose, three examples are presented. These examples have been chosen according to the fact that actual productions have been made. After a description of the Arronax facility, which produces two out of the three isotopes on a regular basis, and intends, in the future, to explore the possibility to produce the last one, each isotope is presented. The added value of using deuterons as projectiles is presented for each of them.

## The Arronax Facility

### The cyclotron

The Accelerator for Research in Radiochemistry and Oncology in Nantes Atlantique (Arronax) (2) cyclotron has turned into operation in February 2011. It accelerates both positive (HH^+^, He^++^) and negative ions (H^−^, D^−^) up to 70 MeV and delivers up to 750 μAe (two beams of 375 μAe) of protons. This has been tested successfully for 24 h in a row during the commissioning phase. Arronax can deliver up to 70 μAe of alpha-particles. The capabilities of the cyclotron are summarized in Table 1.

**Table 1:** **Characteristics of the beams available at Arronax**.

Beam	Accelerated particles	Energy range (MeV)	Intensity (μAe)	Number of simultaneous extracted beams
Protons	H^−^	30–70	<375	2
	HH^+^	Fixed 17	<50	1
Deuterons	D^−^	15–35	<50	2
α-Particles	He^++^	Fixed 68	<70	1

Negative ions are extracted using the stripper foil technique. This technique allows beam extraction with 99% efficiency within a large range of incident energy by changing the radial position of the foil. Here, the proton beam can be extracted from 30 to 70 MeV. Arronax is equipped with two such devices diametrically opposed. This gives the ability to deliver two beams with different energies and intensities (up to 350 μAe each for protons) at the same time. Same feature can be made for the deuteron beam but with a lower intensity (see Table 1).

Positive ions are extracted using an electromagnetic septum. In this case, only one beam output is available at a fixed energy of 68 MeV for alpha-particles (extraction efficiency of 83%) and 17 MeV for HH^+^ ions (extraction efficiency of 80%). This last possibility provides a proton beam at lower energy than that of H^−^ particles without the use of a beam energy degrader.

### The surrounding facility

**Figure 1:**
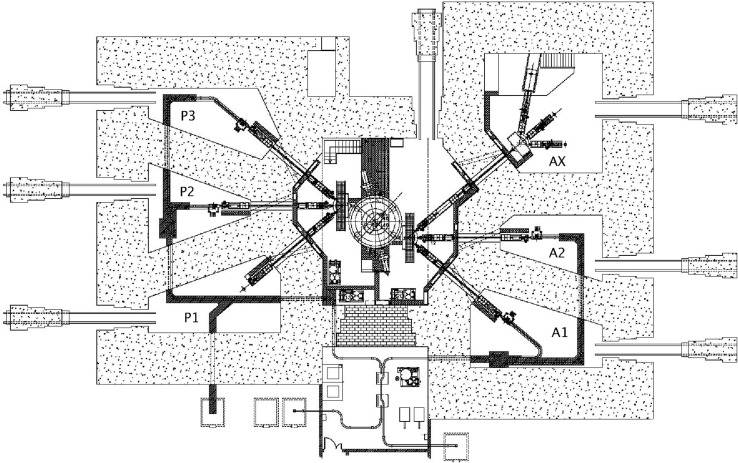
**Schematic view of the ARRONAX cyclotron and its target vaults**.

Arronax can deliver a particle beam in six experimental vaults named AX, A1, A2, P1, P2, and P3 (see Figure 1). Due to the extraction method, which is different for negative and positive ions, protons and deuterons are available in all experimental vaults, whereas alpha-particles and HH^+^ generating protons are available only in vaults A1, A2, and AX.

Vaults A1, A2, P2, and P3, which are dedicated to radionuclide production, are equipped with irradiation stations and remote pneumatic transfer systems (rabbit system) of the irradiated materials from the beam lines into the hot cells. This system insures the vacuum tightness in the beam lines and allows the connection to a water cooling system during irradiation. It can handle different types of targets depending on the desired radionuclide. We have developed two different designs. The first one accommodates a target tilted at 15° with respect to beam axis with one face of the target placed in vacuum and the other one cooled with water. The other type of rabbit can accommodate encapsulated targets placed at 90° with respect to the beam axis, which are water cooled all over their surface. A 4-fingers collimator is placed in front of the target station to center the beam and to limit its diameter.

In vault P1, a high power accelerator-driven neutron source is installed (3).

The largest vault, AX, is devoted to radiochemistry, physics, and radiobiology experiments as well as advanced student training. The beam line entering this vault can be sent in three different sub-beam lines at the end of which experimental devices are placed. One of the beam lines is directed to a pit providing a vertical beam, which is of great interest for the irradiation of chemical solutions and cells for radiolysis or radiobiology experiments. The two other lines are currently used for physics and radiolysis experiments.

In addition, several laboratories (radiochemistry, biochemistry, hot cells, radiolabeling, chemical analysis, nuclear metrology …) are available allowing radiopharmaceuticals development and quality control of final products.

## Deuteron Production of Non-Conventional Radionuclides

To produce alternative isotopes, deuteron beams can, in some cases, be more interesting than proton beams. Three examples are presented below to illustrate this fact. All the nuclear data used were extracted from the NUDAT database (4).

### Scandium-44

Sc-44g has a half-life of 3.97 h. It decays through electron capture in 5.73% of the cases and by β^+^ emission in 94.27% of the cases with a mean kinetic energy of 632 keV. It can be used for PET imaging and will be complementary to the shorter half-life ^68^Ga (*T*_1/2_ = 67.71 min). It is possible to produce an excited state of Sc-44 called Sc-44m (*T*_1/2_ = 58 h), which decays to the ground state by gamma emission (*E*_γ_ = 271.24 keV, *I*_γ_ = 86.7%). It has been proven that when a molecule is labeled with Sc-44m, the Sc-44g formed by its decay remains attached to the molecule (5). This indicates that Sc-44m/Sc-44g may be used as an *in vivo* generator to study long biological processes such as those associated to labeled full antibodies.

Among the scandium isotopes, Sc-47 (β^−^ emitter) is well suited for use in targeted radionuclide therapy. There is then a potential interest to use Sc-44 for dosimetry studies before using Sc-47 for therapy, as a theranostic approach (6).

Finally, associated to Sc-44g decay, a high-energy gamma ray at 1.157 MeV with a high probability of occurrence (99.9%) is emitted within nanoseconds. By measuring the incident direction of this photon and the line of response of the 511 keV photons resulting from the β^+^ decay, it is possible to get, on an event by event basis, the emitter location in three dimensions as proposed by Grignon et al. (7). With a Compton telescope using a new generation of cameras based on a liquid xenon time projection chamber, a spatial resolution of 2.3 mm has been estimated with an injected activity of 0.5 MBq for a Sc-44g point source emitter (7).

**Figure 2:**
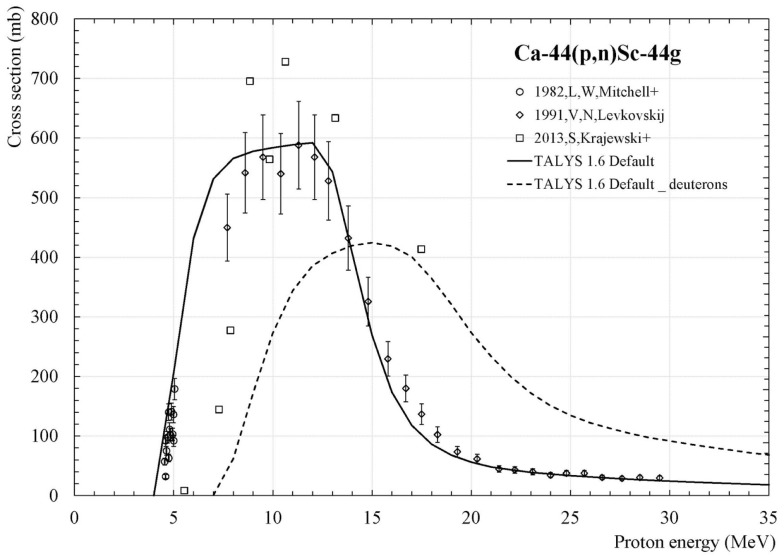
**Sc-44g production cross section for proton and deuteron-induced reaction on Ca-44**. The symbols correspond to the proton data of Levkovskij (8), the full line to TALYS calculation for protons and dashed line to TALYS calculation for deuterons. TALYS 1.6 has been used for these calculations.

Sc-44g and Sc-44m can be produced using enriched ^44^CaCO_3_ as target_._ The use of an enriched target maximizes the scandium production and reduces the production of contaminants. Using protons, production is achieved through the ^44^Ca(p,n) reaction. This reaction has an energy threshold of 4.536 MeV. One set of cross-section data point exists (8) and can be used to estimate the production yield. On Figure 2, the evolution of the production cross-section of Sc-44 as a function of the proton incident energy is presented. Data of Levkovskij (8) have been normalized by a factor 0.8 due to a monitor problem (9). The maximum value of the cross-section is obtained for 12 MeV protons. The production of the Sc-43, via the reaction (p,2n), starts at 14,458 MeV. By selecting proton incident energy below this value, it is possible to prevent the production of Sc-43 although this isotope is an attractive candidate for conventional PET imaging.

As stated before, Sc-44m is also produced during the irradiation. The production cross-section of the metastable state changes with the incident energy of the proton energy. The ratio of the production cross-section of the metastable state over the ground state has been measured by Sachdev et al. (10) for the (p,n) reaction and its values are presented in Table 2. The measurements made by Levkovskij (8) give values of the same order of magnitude. In the energy range of interest, the Sc-44m will represent <15% of the produced Sc-44g + Sc44m atoms. It has to be kept in mind that, experimentally, we have access to the activity and not to the number of produced isotope. Due to the large difference in half lives, activity ratio will be much smaller (divide by a factor of ~15). Another parameter that affects this ratio is the irradiation time. The saturation is reached earlier for Sc-44g than Sc-44m.

**Table 2:** **Evolution of the cross-section ratio as a function of the proton incident energy (10)**.

E (MeV)	6	9	12	15	18	21	24	30	40	50	60	72	85
σ(Sc-44m)/(σ(Sc-44g)	0.015	0.063	0.090	0.140	0.20	0.24	0.23	0.19	0.16	0.14	0.15	0.16	0.17

An alternative production route is to use deuterons as projectiles through the ^44^Ca(d,2n) reaction, which has an energy threshold equal to 6.964 MeV. We did not find cross-section data with deuterons in the literature and we have therefore chosen to use the TALYS code version 1.6 (11) to estimate the production yields in order to compare with the proton route. TALYS is a computer program for simulation of nuclear reactions induced by light projectiles on nuclei heavier than carbon. It integrates many theoretical models that predict the behavior of various observables, among which the production cross-sections, in the energy range from 1 keV to 1000 MeV. TALYS can work with a limited number of input parameters: the type of projectile and its incident energy, the target element and its atomic mass. Default settings are assigned to each nuclear model and several parameters can be modified to better describe the data. In this work, we used the default settings.

Before using the code to obtain the (d,2n) cross-section values, we have compared the experimental cross-section for the reaction (p,n) measured by Levkovskij (8) with the values predicted by TALYS. The data are presented as symbols on Figure 2 whereas the TALYS values are presented as full lines on the same Figure. A good agreement is found between the data and the code values giving us confidence on the ability of this code to predict deuteron cross-section values.

TALYS was then used to estimate the Sc-44g and Sc-44m production cross-sections associated to the (d,2n) reaction on Ca-44. On Figure 2, the dashed line corresponds to the Sc-44g cross-section obtained with the TALYS code. We find that the production cross-section maximum value with deuterons is comparable to the proton one. The curve has a maximum a little lower in amplitude but it is wider. Based on this curve, we see that we must be in the energy range 10–20 MeV to optimize the production of Sc-44g with deuterons. This range is consistent with the available deuteron energy on ARRONAX. In addition, production being related to the integral of cross-section over energy, yield using deuterons will be at least equal to that obtained with protons.

**Figure 3:**
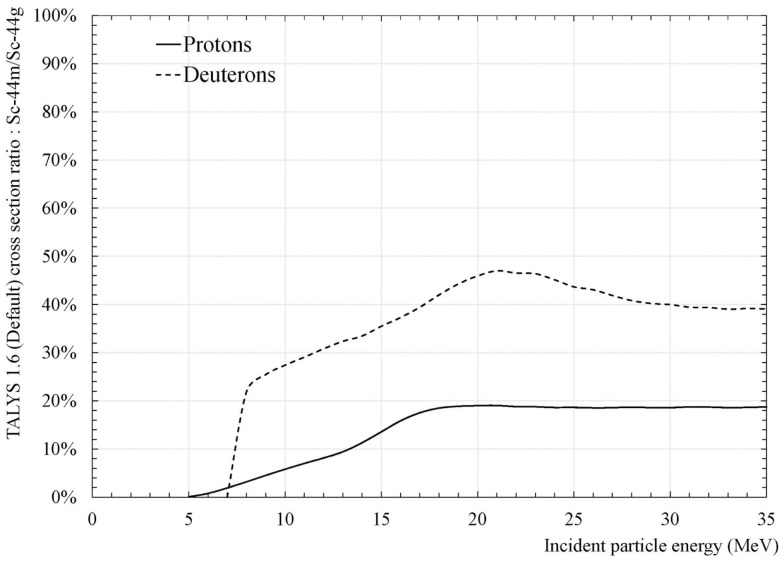
**Ratio of the cross section associated to Sc-44m over the cross-section associated to Sc-44g obtained from the TALYS code**.

TALYS can also calculate the cross-section ratio of Sc-44m over Sc-44g. The latter is presented as a function of the projectile incident energy in Figure 3. TALYS calculations for protons and deuterons are presented, respectively, as full line and dashed line. From this figure, it is clear that the values obtained with deuterons are larger than with protons. With deuterons, as much as three times more Sc-44m can be expected as compared to protons.

The last point of interest, when comparing both production routes, is related to the co-production of other isotopes. Using a proton beam and a typical enriched target, only Sc-43 will have an impact on the specific activity. The other produced isotopes are stable or have short periods (less than minutes). Sc-43 amount can be tuned by changing the proton incident energy. Below the (p,2n) reaction threshold, no Sc-43 is produced.

Using a deuteron beam and a typical enriched target, Ca-45 (*T*_1/2_ = 162.61 days) will be produced via (d,p) reaction as well as K-42 (*T*_1/2_ = 12.321 h) via the (d,α) and K-43 (*T*_1/2_ = 22.3h) via the (d,2pn) reactions. These isotopes can be removed during the chemical separation process but they will put some constraints on it. The potassium isotopes produce scandium isotopes during their decay that will have an impact on the specific activity of the final product. It is then necessary to speed up the chemical process in order to keep a high-specific activity.

At Arronax, small amounts of Sc-44g and Sc-44m have been made using an irradiation station devoted to the production of low activity batches (a few millicurie at maximum). With this device, our target is placed in air, 6.6 cm downstream the beam line output closed by a 75 μm-thick kapton foil, which makes the separation between the vacuum in the line and the air in the vault. Each target is made of 500 mg of ^44^CaCO_3_ pressed to form an 18 mm diameter pellet. To prevent any contamination from the CaCO_3_ powder during irradiation, each target is placed inside a frame made of 75 μm kapton sheets on both sides. An original chemistry has been developed using DGA chromatographic column (5) from TrisKem International SAS (France). After the chemistry, the expensive target material is recovered for reprocessing.

The target is generally irradiated with deuterons of 16.4 MeV for 180 min with a beam intensity around 300 nA. Scandium is recovered as ScCl_3_. Typical activity for Sc-44g is 90 MBq whereas it is 1.8 MBq for Sc-44m at the end of the chemistry step (around 4 h after EOB). The activity ratio is then equal to 2%, which is coherent with TALYS calculations. If one except Sc-44g and Sc-44m, all radionuclides are below the detection limit using our HP-GE detector. Using an ICP-OES, the main stable contaminants have been identified (Al, Fe, Zn, and Sc) and the total content is lower than 1 ppm. These numbers show that deuterons can be advantageously used to produce Sc-44m with a high-specific activity.

### Copper-64

Cu-64 has a half-life of 12.7004 h. It decays through electron capture in 44.00% of the case, by β^−^ in 38.48% with a maximum energy of 579.4 keV and finally by β^+^ emission in 17.52% of the case with a maximum energy of 653 keV. Associated to these emissions, there is a high-energy gamma ray at 1345.75 keV with a low probability of occurrence (0.4749%). Considering the emitted radiations, it can be used for both therapy and PET imaging.

**Figure 4:**
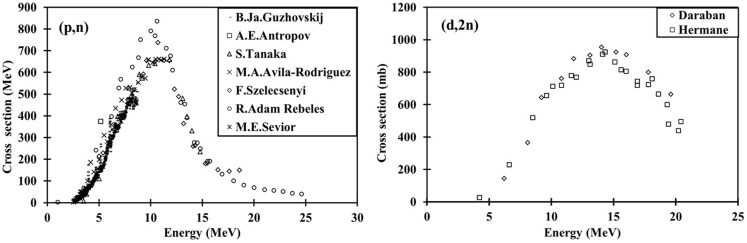
**Production cross-section as a function of the projectile energy for Ni-64(p,n)Cu-64 (left) and Ni-64(d,2n)Cu-64 (right)**. Inspired by Ref. (17).

Several routes to produce Cu-64 have been reported in the literature (12–16) among which the irradiation of enriched Ni-64 with protons is the most often used. As most (p,n) reactions, production cross-sections are high (see left plot on Figure 4). Looking at the deuteron production route, it is found that the maximum cross-section value for (d,2n) reaction is higher than for (p,n), reaching 800 mb at 14 MeV. In addition, the curve evolves more slowly around the maximum for (d,2n) in comparison to (p,n) indicating a possible higher yield using deuterons (17). Using a 16 MeV deuteron beam, it is possible to take full advantage of the maximum cross-section. In this condition, theoretical production yield has been calculated and compared with that obtained for a 12 MeV proton beam (see Table 3). It shows that it is possible to produce the same amount of Cu-64 in both cases with a target 25% thinner using deuterons.

**Table 3:** **Cu-64 calculated yields for (p,n) and (d,2n) production routes**.

Nuclear reaction	Energy range (MeV)	Calculated yield (MBq/μAh)	Target thickness (μm)	Target thickness at 15°(μm)
Ni-64(p,n)Cu-64	12–9	228	120	31.05
Ni-64(d,2n)Cu-64	16–13	206	90	23.29

Differences in the co-produced contaminants exist between the two production routes. On the one hand, the deuteron beam produces Ni-65 (*T*_1/2_ = 2.52 h) and stable Cu-65, which are not created at all with the (p,n) reaction. On the other hand, less Co-61 (*T*_1/2_ = 1.65 h), which is created using proton via (p,α), is produced with the deuteron reaction. When using a deuteron beam, the production of Ni-65 (which decays to Cu-65) and Cu-65 will have an impact on the final specific activity. In order to have some hints on their contributions, production yields have been calculated for these isotopes and for Cu-63. When available, data have been taken from Kinsey et al. (4). Otherwise, we have used the TALYS code with default parameters (11) to get cross-section values. In Table 4, the calculated yield values are presented for a 1 h irradiation at 1 μA.

**Table 4:** **Direct and indirect productions of stable copper isotopes with a deuteron beam on Ni-64**.

Isotope	Half-life	Decay product	Expected yield (EOB) atoms
^65^Ni	2.52 h	^65^Cu	2.33 E + 12
^63^Cu	Stable	–	2.07E + 11
^65^Cu	Stable	–	6.1 E + 11
	^65^Ni decays during irradiation		3,3 E + 11
^64^Cu	12.7 h	Ni-64	1.31 E + 13

The main stable copper contribution comes from the decay of Ni-65 either during irradiation or after. The latter contribution is strongly correlated to the time of the chemistry, the shorter the separation is, the lower the contribution is, whereas the first contribution increases with the irradiation time.

By considering the worst case in which all the Ni-65 has decayed to Cu-65 when the chemistry is performed, we end up with 3.8 atoms of Cu-64 for 1 atom of cold copper coming for a 1 h irradiation. This corresponds to a specific activity of 7.22 GBq/nMol as compared to the theoretical value for proton, which is 9.13 GBq/nMol. These numbers are far above what is necessary for antibody labeling.

At our facility, the deuteron production route is used for Cu-64 production since Arronax is able to deliver this type of particles (see Table 3). A highly enriched (>99%) Ni-64 target electroplated on a gold backing (99.99% purity) is used and an original chemistry has been developed using HBr on AG1X8 chromatographic column. After the chemistry, the expensive target material is recovered (recovery yield around 95%) for reprocessing.

A typical irradiation is carried out with 50 μA deuterons beam at 16 MeV impinging a 10 μm-thick target for 150 min. Copper-64 is recovered as CuCl_2_ with a high-radioisotopic purity (>99.90%). Using an ICP-OES, the main contaminants have been identified as Ni, Cu, and Fe. On a routine basis, we produce 12 GBq at the end of bombardment. Production can be increased by using a thicker target and increasing the irradiation duration. These numbers show that deuteron can be advantageously used to produce Cu-64.

### Rhenium-186g

Rhenium is a Group *7* congener of Tc and therefore, in many cases, shares remarkably similar chemical behavior to Tc. Thus, Tc-99m radiocomplexation knowledge can often be applied to rhenium radionuclides. Re-186g is a β^−^ emitter with a 137.157 keV γ emission (branching ratio of 9.42%) that can be used for imaging with SPECT. Its half-life is 3.7183 days. There is another β^−^ emitter isotope, ^188^Re, with a half-life of 0.7 days, which has a 155.032 keV γ emission suitable for imaging. In addition to their different half lives, the mean β^−^ energy of these two rhenium isotopes is different: 347 and 763 keV for Re-186g and Re-188, respectively. Because the chemistry of rhenium is close to that of technetium, Tc-99m/Re-186g and Tc-99m/Re-188 can act as potential theranostic pairs.

Re-188 can be obtained with a high-specific activity through a W-188/Re-188 generator. Unfortunately, W-188 requires a double neutron capture to be produced limiting its production to high-flux nuclear reactor. Using nuclear reactors, it is not possible to get high specific activity Re-186g. However, it is possible to use accelerator to that purpose.

**Figure 5:**
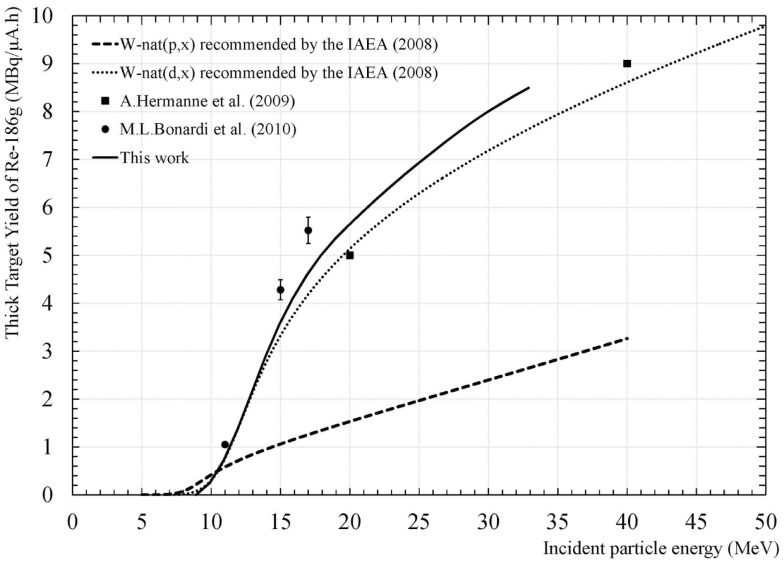
**Re-186g thick-target yield obtained from IAEA (20) data with proton and deuteron and value determined from new experimental data from Duchemin et al. (18)**. Symbols correspond to values extracted from Hermann et al. (21) and Bonardi et al. (22).

A promising production route is the W-186(d,2n). Its threshold energy is equal to 3.6 MeV. Deuteron with incident energy below 17.6 MeV can be used to produce high-radionucleidic purity and high-specific activity of Re-186g. Using deuterons as projectiles, five times more Re-186g can be produced as compared to protons as projectiles (see Figure 5). Based on the calculation of Duchemin et al. (18), using an enriched W-186 target and an energy range of 3.5–17.6 MeV, a thick-target yield of 16.8 MBq/μA⋅h can be obtained, which corresponds to 0.5 Ci (EOB) for 24 h irradiation at 50 μA. These numbers are in agreement with data from Xiaodong et al. (19), which have reported the production of Re-186g using a 16 MeV deuteron beam via the (d,2n) reaction on isotopically enriched W-186 metal powder. After chemical extraction and purification, no-carrier-added Re-186g saline solution was obtained with a radionuclidic purity >99.9%. Main isotopic impurities were Re-183 and Re-184g. The experimental thick-target yield of Re-186g was determined to be ~529 μCi/μA⋅h. These numbers show that deuteron is the projectile of choice to produce high-specific activity of Re-186g.

## Conclusion

Recently, the theranostic paradigm has renewed the interest for unconventional radionuclides in nuclear medicine. Several radioisotopes with different properties (half-life, beta energy, gamma emissions) are under study for either imaging or therapeutic use. In some cases, the use of deuterons as projectiles to produce these radionuclides can be beneficial. Scandium-44 can be used for imaging. In addition to the ground state (Sc-44g), there is a metastable state (Sc-44m) that can be used as an *in vivo* Sc-44m/Sc-44g generator. When using deuterons to produce these isotopes, one maximizes the production of the metastable state as compared to the ground state, which is of interest if one wants to use Sc-44 for imaging for long processes. Another example is the production of Cu-64. It can be produced from deuteron irradiation using the same target as for proton but using a lower quantity of Ni-64, which is a very expensive material. A theoretical study shows that the specific activity is a little lower than in the case of proton irradiation but is still high enough for medical applications. Finally, in the case of Re-186, the use of deuterons is the only viable production route since the production yield is five times higher than that obtained using protons.

At the Arronax facility, we have access to deuteron beam at the right energy and use it already for routine production of Cu-64 and Sc-44m/Sc-44g. We have started to look at the Re-186g production with deuterons.

## Conflict of Interest Statement

The authors declare that the research was conducted in the absence of any commercial or financial relationships that could be construed as a potential conflict of interest.
